# Chromosomal genes modulating fosfomycin susceptibility in uropathogenic *Escherichia coli*: a genome-wide analysis

**DOI:** 10.1128/aac.01417-24

**Published:** 2025-02-25

**Authors:** Yibing Ma, Mattia Pirolo, Lily Giarratana, Karen Leth Nielsen, Susanne Häussler, Luca Guardabassi

**Affiliations:** 1 Department of Veterinary and Animal Sciences, University of Copenhagen 545332 , Frederiksberg, Denmark; 2 Department of Clinical Microbiology, Rigshospitalet, København Ø, Denmark; 3 Department of Molecular Bacteriology, Helmholtz Centre for Infection Research, Braunschweig, Germany; Universita degli studi di roma La Sapienza, Rome, Italy

**Keywords:** *E. coli*, fosfomycin, TraDIS

## Abstract

*Escherichia coli* acquires fosfomycin resistance through chromosomal mutations that reduce drug uptake and by drug-inactivating enzymes. However, the complete resistance mechanisms remain to be elucidated. The aim of this study was to elucidate the genetic mechanisms regulating fosfomycin susceptibility in uropathogenic *E. coli* (UPEC). We constructed a highly saturated transposon mutant library containing >340,000 unique Tn5 insertions in a clinical UPEC strain. We conducted transposon-directed insertion site sequencing (TraDIS) to screen for chromosomal genes whose mutations are beneficial for bacterial growth and survival in the presence of fosfomycin at 4 and 32 µg/mL. TraDIS analysis identified 67 genes including known resistance determinants (*n* = 13) as well as a set of novel genes modulating fosfomycin susceptibility (*n* = 54). These genes are involved in pyruvate metabolism, pentose phosphate pathway, nucleotide biosynthesis, DNA repair, protein translation, cellular iron homeostasis, and biotin biosynthesis. Deletion of 16 selected genes in the wild-type strain resulted in growth advantages and decreased susceptibility when exposed to fosfomycin. Notably, deletion of DNA repair genes (i.e.*, mutL* and *mutS*) and purine synthesis genes (i.e., *purB* and its upstream gene *hflD*) led to the most significant advantages in competitive and non-competitive growth in the presence of fosfomycin, as well as the highest increase of fosfomycin MIC (8- to 16-fold). These findings provide a genome-wide overview of genes required for maintaining fosfomycin susceptibility in *E. coli*, highlighting new mutations and functional pathways that may be used by UPEC to develop clinical resistance.

## INTRODUCTION

Fosfomycin is one of the first-line treatment options for uncomplicated urinary tract infection (1, 2). This antibiotic targets MurA, the enolpyruvate transferase responsible for catalyzing the first committed step in peptidoglycan assembly, irreversibly inhibiting cell wall biosynthesis and ultimately leading to cell death (3). Fosfomycin uptake depends on two nutrient transport systems, namely, GlpT and UhpT, whose expression is induced by their respective substrates, glycerol-3-phosphate (G3P), and glucose-6-phosphate (G6P), and requires the second messenger, cyclic adenosine monophosphate (cAMP) (4). UhpT expression is additionally under the regulation of a pathway encoded by the uhpABC operon (4, 5). Reduced uptake and drug inactivation are the main resistance mechanisms, while resistance due to mutations or overexpression of the target protein is infrequently reported in clinical isolates (4, 6). Reduced uptake has been associated with mutations in genes involved in the GlpT and UhpT transport systems (i.e., *glpT*, *uhpT*, *uhpABC*) or required for cAMP regulation (i.e., *cyaA*, *crp*, *crr,* and *ptsHI*) (4, 5, 7–9). Drug inactivation is caused by a variety of fosfomycin-modifying enzymes (FosA, FosB, FosC, and FosX), which are further classified into subtypes based on amino acid sequence (4, 6). FosA is the most frequently reported enzyme in Enterobacteriaceae with FosA3 being the most common variant, especially among extended-spectrum beta-lactamase (ESBL)-producing *Escherichia coli* isolates from patients and animals (10, 11).

The known fosfomycin resistance determinants are sometime undetected in clinical isolates and in spontaneous mutants observed upon disk diffusion testing, indicating that the mechanisms of resistance to this antibiotic are not yet fully understood (12, 13). This knowledge gap is confirmed by the presence of non-susceptible subpopulations within susceptible strains, a phenomenon long attributed to loss-of-function mutations in the *uhp* genes (14). A recent study sequenced a limited number of inner colonies within the inhibition zone of fosfomycin and analyzed a small proportion of non-*uhp* mutations, identifying a few novel loci whose mutations contributed to reduced susceptibility (15). Another recent study employed transposon mutagenesis combined with next-generation sequencing in a laboratory *E. coli* strain, revealing various loci involved in fosfomycin susceptibility, including genes that mediate glucose metabolism, phosphonate catabolism, and phosphate import (7). Here, we used the same genome-wide approach, Transposon Directed Insertion-site Sequencing (TraDIS), to identify chromosomal non-essential genes that modulate fosfomycin susceptibility in a clinical uropathogenic *E. coli* (UPEC). Our objective was to validate the findings of the previous TraDIS study in a clinical strain. We identified a diverse set of chromosomal genes whose inactivation enhanced growth and viability of the UPEC strain in the presence of fosfomycin, demonstrating that deletion of these genes reduced susceptibility to the antibiotic.

## RESULTS

### Density of the transposon mutant library

We constructed a transposon mutant library in a fosfomycin susceptible (MIC = 0.5 µg/mL) UPEC strain (strain ID FREC5), yielding >300,000 individual mutants. Sequencing of the library with two biological replicates generated >12 million reads. As depicted in Fig. S1, mapping reads to the genome of FREC5 led to identification of >340,000 unique transposon insertions including 274,798 insertions present in CDS regions. This indicates an average of one transposon insertion per 14 nucleotides among the genome, confirming the saturation of the library. Gene essentiality analysis revealed that 406 genes (9% of the genome) are essential for the growth of FREC5 under our experimental conditions (see Materials and Methods).

### Growth patterns of the mutant library exposed to fosfomycin

We compared the growth curve of the mutant library with that of the FREC5 wild-type (WT) strain under exposure to a wide range of fosfomycin concentrations (1–256 µg/mL). As shown in Fig. S2, the mutant library displayed growth curves similar to the WT strain at 0–16 µg/mL of fosfomycin but drastically differed from the WT at higher concentrations. Notably, the mutant library exposed to 32 µg/mL of fosfomycin exhibited growth advantages compared to that exposed to 4 µg/mL of fosfomycin after 6 h of incubation, and the cell density reached the same level as that observed in the absence of fosfomycin after 24 h. These results indicated that mutants with growth advantages were selected by 32 µg/mL of fosfomycin. Accordingly, this concentration was used to identify the mutants in the following TraDIS experiment.

### Identification of genes whose mutations benefit growth in the presence of fosfomycin

The contribution of gene mutations to selective advantages was evaluated by measuring enrichment of transposon insertion frequencies following exposure to 4 and 32 µg/mL of fosfomycin, respectively. Genes exhibiting a fourfold or greater increase in mutant frequencies [log2 fold change (logFC) ≥2 and FDR-adjusted *P*-value (*q*-value) ≤0.05] following fosfomycin exposure at 32 µg/mL compared to 4 µg/mL or 0 µg/mL, as well as 4 µg/mL compared to 0 µg/mL, were selected. These genes were further examined on their transposon insertion profile to exclude those containing high sequencing read counts on a single insertion site (Fig. S3). This analysis led to the identification of genes with transposon insertion frequencies significantly enriched at 32 µg/mL compared to 4 µg/mL (*n* = 55), 32 µg/mL compared to 0 µg/mL (*n* = 78), and 4 µg/mL compared to 0 µg/mL (*n* = 40) (Fig. S4 and Supplementary Data set). A total of 67 overlapping genes were grouped into nine function categories including fosfomycin transport (*n* = 5), cAMP regulation (*n* = 5), pyruvate metabolism (*n* = 8), pentose phosphate pathway (*n* = 4), nucleotide biosynthesis (*n* = 6), DNA repair (*n* = 7), protein translation (*n* = 6), cellular iron homeostasis (*n* = 9), and biotin biosynthesis (*n* = 6), while 11 genes with other functions were not assigned to any of these categories (Table 1). Among these genes, 13 were previously associated with fosfomycin resistance, including the fosfomycin transport genes (i.e., *glpT*, *uhpT,* and *uhpABC*) (4, 5, 7–9), cAMP-related genes (i.e., *ptsHI*, *cyaA*, *crp,* and *crr*) (4, 5, 7–9), *pykF* (16), *ackA* and *pta* (17). Such genes were excluded from further analysis.

**TABLE 1:** List of inactivated genes in mutants that were significantly enriched after exposure of *Escherichia coli* FREC5 to fosfomycin at 32 µg/mL compared to 0 µg/mL

Gene^*a*^^,*b*^	Product	logFC	*q*-value
Fosfomycin transport			
*glpT*	Glycerol-3-phosphate transporter	19.17	4.64E−20
*uhpT*	Hexose-6-phosphate:phosphate antiporter	8.96	4.69E−08
*uhpB*	Sensor histidine kinase UhpB	7.09	5.45E−06
*uhpA*	DNA-binding transcriptional activator UhpA	6.17	3.59E−05
*uhpC*	Inner membrane sensor protein UhpC	6.14	5.76E−05
PTS and cAMP regulation			
*ptsI*	Phosphoenolpyruvate-protein phosphotransferase	18.79	3.9E−17
*ptsH*	Phosphocarrier protein HPr	18.00	2.49E−14
*crp*	cAMP-activated global transcriptional regulator CRP	17.35	3.25E−15
*cyaA*	Adenylate cyclase	14.21	2.7E−14
*crr*	PTS system glucose-specific EIIA component	13.23	6.67E−12
Glycolysis and pyruvate metabolism			
*pykF*	Pyruvate kinase 1	15.03	3.25E−15
*ackA*	Acetate kinase	10.13	3.71E−09
***aceF***	Pyruvate dehydrogenase E2 subunit	8.69	7.34E−08
*aceE*	Pyruvate dehydrogenase E1 subunit	8.75	4.69E−08
*pta*	Phosphate acetyltransferase	7.84	2.29E−07
***pdhR***	Pyruvate dehydrogenase complex repressor	7.72	2.97E−07
*acnB*	Aconitate hydratase B	5.15	6.98E−05
*pgi*	Glucose-6-phosphate isomerase	5.00	0.00026
Pentose phosphate pathway			
***rpe***	Ribulose-phosphate 3-epimerase	10.65	1.83E−09
***tktA***	Transketolase 1	10.60	1.08E−09
*pgl*	6-Phosphogluconolactonase	7.40	5.85E−07
*gnd*	6-Phosphogluconate dehydrogenase	3.82	0.007945
Nucleotide biosynthesis			
***purB***	Adenylosuccinate lyase	15.21	6.91E−14
***hflD***	High frequency lysogenization regulator (upstream of *purB*)	12.98	1.39E−12
*dcd*	dCTP deaminase	8.71	7.34E−08
*cmk*	Cytidylate kinase	8.02	6.23E−07
*hpt*	Hypoxanthine phosphoribosyltransferase	6.28	4.85E−06
*udk*	Uridine kinase	6.04	2.26E−05
DNA replication and repair			
*rep*	ATP-dependent DNA helicase Rep	9.46	7.89E−09
*mutT*	8-oxo-dGTP diphosphatase	8.54	5.06E−06
***mutL***	DNA mismatch repair protein MutL	8.20	1.98E−07
*topA*	DNA topoisomerase 1	8.44	9.14E−08
***mutS***	DNA mismatch repair protein MutS	7.52	1.21E−06
*mutH*	DNA mismatch repair protein MutH	6.90	5.06E−06
*recC*	RecBCD enzyme subunit RecC	4.62	3.38E−06
rRNA processing, ribosome, and translation			
*rpmF*	50S ribosomal protein L32	10.04	2.82E−08
***yceD***	Large ribosomal RNA subunit accumulation protein YceD	9.68	7.89E−09
***rng***	Ribonuclease G	9.21	1.68E−08
*nusA*	Transcription termination/antitermination protein NusA	8.87	2.81E−08
*tufB*	Elongation factor Tu 2	7.71	3.31E−07
*tufA*	Elongation factor Tu 1	6.57	5.45E−06
Iron-sulfur cluster and ferric homeostasis			
*iscU*	Iron-sulfur cluster assembly scaffold protein IscU	10.95	0.000922
***hscB***	[Fe-S] cluster biosynthesis co-chaperone protein HscB	10.54	4.52E−08
***fur***	Ferric uptake regulation protein	10.20	3.71E−09
*fdx*	2Fe-2S ferredoxin	9.83	7.69E−08
*hscA*	Chaperone protein HscA	8.88	3.73E−08
*fepG*	Ferric enterobactin transport system permease protein FepG	5.33	1.49E−07
*fepD*	Ferric enterobactin transport system permease protein FepD	5.11	7.61E−06
*fepB*	Ferrienterobactin-binding periplasmic protein	5.09	3.46E−06
*fepC*	Ferric enterobactin transport ATP-binding protein FepC	5.06	1.93E−06
Biotin biosynthesis			
***bioC***	Malonyl-[acyl-carrier protein] O-methyltransferase	6.18	1.92E−05
***bioD***	ATP-dependent dethiobiotin synthetase BioD	6.27	1.41E−05
*bioB*	Biotin synthase	5.96	2.95E−05
*bioF*	8-Amino-7-oxononanoate synthase	5.88	2.98E−05
*bioA*	Adenosylmethionine-8-amino-7-oxononanoate aminotransferase	5.76	4.4E−05
*bioH*	Pimeloyl-[acyl-carrier protein] methyl ester esterase	5.12	0.000401
Other functions			
***plsX***	Phosphate acyltransferase	7.85	5.15E−05
***fruK***	1-Phosphofructokinase	8.49	1.64E−05
*galT*	Galactose-1-phosphate uridylyltransferase	7.62	1.42E−06
*uxuR*	DNA-binding transcriptional repressor UxuR	6.86	0.0003
*gmhB*	D-glycero-beta-D-manno-heptose-1,7-bisphosphate 7-phosphatase	6.56	0.0004
*prc*	Tail-specific protease	5.50	7.19E−05
*bamB*	Outer membrane protein assembly factor BamB	5.01	0.0071
*amiC*	N-acetylmuramoyl-L-alanine amidase AmiC	4.91	0.0009
*tolR*	Tol-Pal system protein TolR	4.65	0.0002
*surA*	Chaperone SurA	3.80	0.0100
*waaQ*	Lipopolysaccharide core heptosyltransferase WaaQ	3.45	0.0245

^*a*^ Genes underlined were previously correlated to fosfomycin susceptibility.

^*b*^ Genes highlighted in bold were selected for constructing deletion mutants.

### Growth of gene-deletion mutants in the presence of fosfomycin

Gene-deletion mutants of FREC5 were constructed to test their ability to grow in the absence or presence of fosfomycin. Genes related to known fosfomycin resistance mechanisms (see above) were excluded, along with genes that displayed no transposon insertions within certain regions of their CDS (e.g., *rpmF* and *iscU*), since their inclusion would lead to potential technical difficulties for deletion. From the remaining genes, we selected the two most significant genes from each functional category based on the highest logFC values, resulting in a total of 16 genes (Table 1).

**Fig 1:**
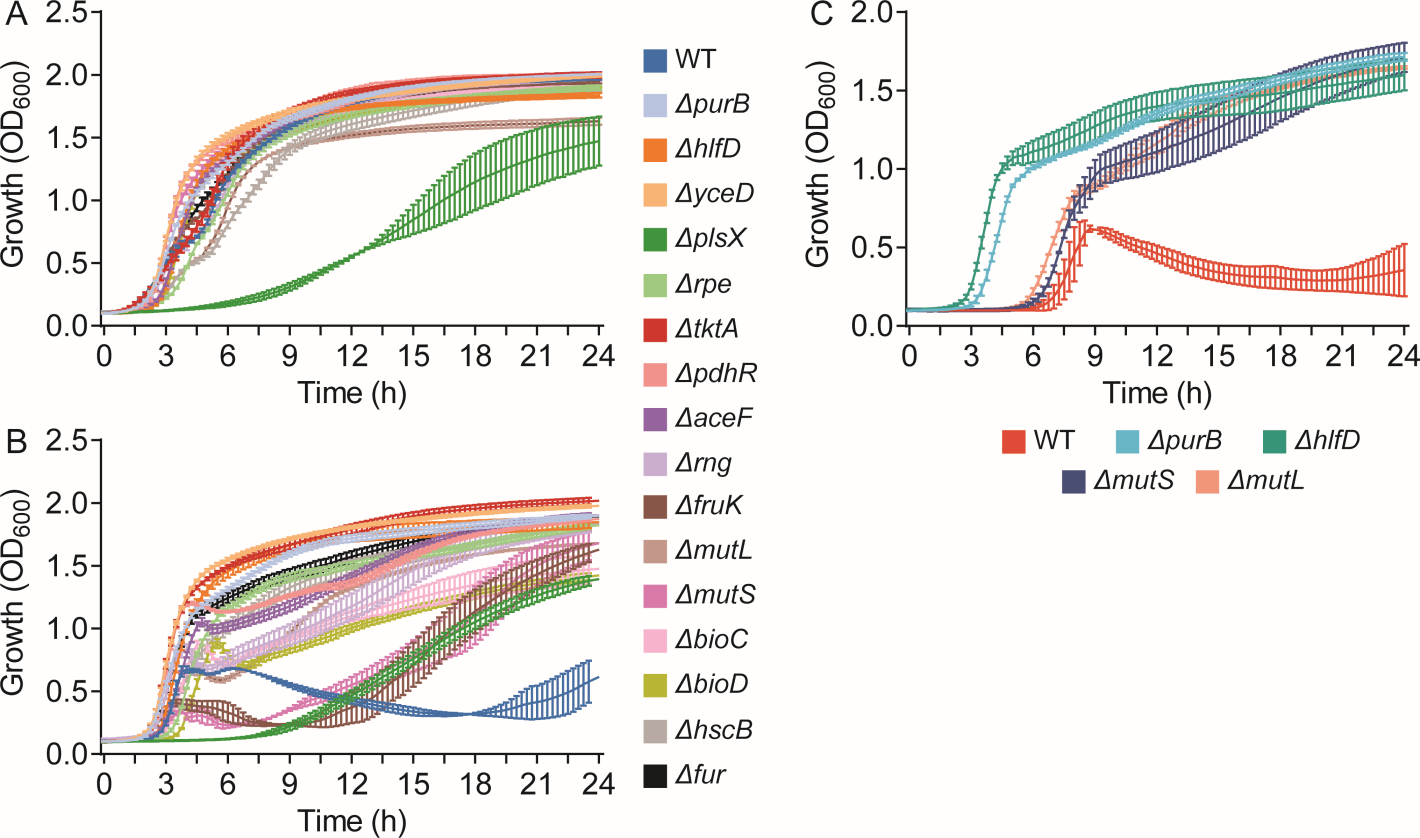
Growth curves of the wild-type (WT) strain and gene-deletion mutants of FREC5 in MHB media supplemented with 0 µg/mL (**A**), 4 µg/mL (**B**), or 32 µg/mL (**C**) of fosfomycin. Growth (OD_600_) was measured at every 15 min in 24 h. Three biological replicates were included for each sample, and data represent the mean ± standard deviation of the triplicates.

In the absence of fosfomycin, Δ*plsX* had a slower growth rate, while the other 15 mutants showed growth curves comparable to the WT strain (Fig. 1A). In the presence of 4 µg/mL of fosfomycin, all the mutants reached cell densities three- to fourfold higher than that of the WT strain after 24 h (Fig. 1B). When exposed to 32 µg/mL of fosfomycin, four mutants, namely, Δ*hflD*, Δ*purB*, Δ*mutL,* and Δ*mutS*, exhibited significant growth advantages compared to the WT strain (Fig. 1C). However, similar to the WT strain, Δ*mutL* and Δ*mutS* also displayed prolonged lag phase of approximately 3 h. The remaining mutants had impaired growth similar to the WT strain (data not shown).

**Fig 2:**
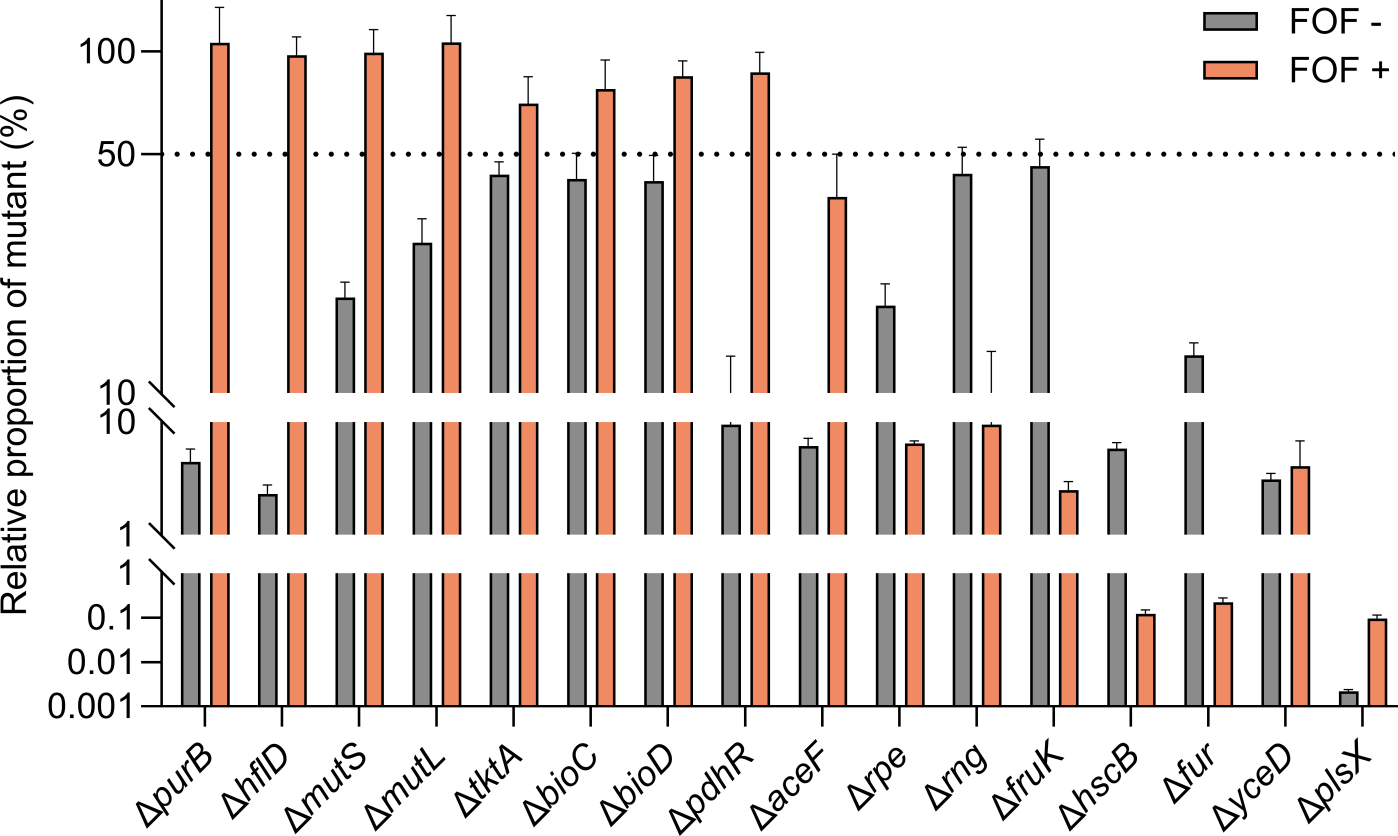
Fitness competition between gene-deletion mutants and the wild-type (WT) strain of FREC5 in the absence and presence of 32 µg/mL of fosfomycin (FOF). Relative mutant proportion represents the percentage of the mutant among the entire bacterial population after 24 h of co-culture with the WT strain. Mutants with a relative proportion higher than 50% were considered to have fitness advantage over the WT strain. Three biological replicates were included for each sample, and data represent the mean ± standard deviation of the triplicates.

In addition, we tested the growth of each mutant in competition with the WT strain. In the absence of fosfomycin, all 16 mutants exhibited relative proportion ranging from 0.002% to 44% after 24 h of co-culture with the WT strain, indicating a variable fitness cost associated with the specific gene deletions. However, in the presence of 32 µg/mL of fosfomycin, eight mutants (i.e., Δ*purB*, Δ*hflD*, Δ*mutS*, Δ*mutL*, Δ*tktA*, Δ*bioC*, Δ*bioD*, and Δ*pdhR*) outcompeted the WT strain, with relative proportion exceeding 50% (Fig. 2). Limited to the four mutants showing the highest relative proportion, namely, Δ*purB*, Δ*hflD*, Δ*mutS,* and Δ*mutL*, we further evaluated their fitness advantages over the WT strain by repeating the 24 h growth competition experiments with measurements at multiple time points. Δ*purB* and Δ*hflD* displayed comparable fitness at each time point, regardless of fosfomycin exposure. Δ*mutS* and Δ*mutL* were both affected by lower fitness cost compared to Δ*purB* and Δ*hflD* in the absence of fosfomycin. In the presence of fosfomycin, however, Δ*mutL* displayed a higher fitness advantage than Δ*mutS* over the WT strain (Fig. S5).

### Antimicrobial resistance phenotype in gene-deletion mutants

To further verify the decreased fosfomycin susceptibility of mutants, we performed MIC testing on Mueller-Hinton agar (MHA) supplemented with 25 µg/mL of G6P, which is recommended by both EUCAST and CLSI to ensure sufficient fosfomycin uptake and experimental reproducibility (18, 19). A 2- to 16-fold increase of fosfomycin MIC was observed in the mutants compared to the WT strain upon supplementation with G6P (Table 2). Consistent with growth curve results, Δ*mutL* and Δ*mutS* displayed the highest MIC (8 µg/mL) with a 16-fold increase compared to the WT strain (0.5 µg/mL), followed by Δ*hflD* and Δ*purB* (4 µg/mL). To exclude the potential effect of G6P on individual gene’s contribution to fosfomycin susceptibility, MICs were also tested on MHA alone, revealing a 2- to 8-fold increase in the mutants, among which Δ*hflD*, Δ*purB,* and Δ*aceF* exhibited the highest MIC (512 µg/mL) (i.e., eightfold increase compared to WT). As expected, fosfomycin MICs significantly increased in either the WT strain or the mutants when tested without G6P compared to those tested with G6P, suggesting that fosfomycin uptake is minimal in the absence of G6P.

**TABLE 2:** Fosfomycin MICs (µg/mL) in the wild-type (WT) FREC5 strain and its derivative gene-deletion mutants tested with or without supplementation of 25 mg/L glucose-6-phosphate (G6P)

Genotype	MIC with G6P	MIC without G6P
FREC5 WT	0.5	64
Δ*mutL*	8	256
Δ*mutS*	8	128
Δ*hflD*	4	512
Δ*purB*	4	512
Δ*plsX*	2	256
Δ*pdhR*	2	256
Δ*aceF*	2	512
Δ*rng*	1	256
Δ*rpe*	1	128
Δ*tktA*	1	256
Δ*yceD*	1	256
Δ*bioC*	1	256
Δ*bioD*	1	256
Δ*hscB*	1	256
Δ*fur*	1	128
Δ*fruK*	1	128

We further investigated whether these mutations conferred resistance to other antimicrobials used for treatment of UTI, namely, amoxicillin, trimethoprim, ciprofloxacin, gentamicin, and azithromycin, without observing any significant increase in the MICs (>2-fold) of the mutants compared to the WT strain (Table S1). On the contrary, specific mutations increased antibiotic susceptibility: Δ*hflD* and Δ*purB* exhibited a fourfold decrease in the MIC of trimethoprim and gentamicin; Δ*bioC* and Δ*bioD* showed an eightfold decrease of gentamicin MIC; and Δ*fur* displayed a fourfold decrease in the MIC of azithromycin.

The enrichment of transposon insertions in the *hflD-purB* operon at 32 µg/mL of fosfomycin (Fig. S6) and the significant increase in the fosfomycin MIC in Δ*hflD* and Δ*purB* (Table 2) prompted us to further investigate their individual role in reducing fosfomycin susceptibility by gene complementation with pACYC184 vector expressing *hflD* or *purB* in both mutant strains. Notably, complementation of *purB* but not *hflD* restored the fosfomycin MIC and the growth phenotype of the WT strain in both Δ*purB* and Δ*hflD* (Table S2; Fig. S7).

## DISCUSSION

This study provides a genome-wide overview of the gene mutations selected in UPEC by different concentrations of fosfomycin. We confirmed the known fosfomycin resistance mechanisms, showing that gene mutations interfering with cAMP (i.e., *cyaA*, *crr*, *crp*, *ptsHI*) or the UhpT and GlpT transporters (i.e., *uhpT*, *glpT*, *uhpABC*) generally confer selective advantages in the presence of fosfomycin (Fig. S4). Interestingly, increased selective advantages under higher fosfomycin stress (i.e., 32 µg/mL) compared to lower stress (i.e., 4 µg/mL) were observed only in the cAMP mutants but not in the transporter mutants, possibly due to the biological costs caused by the *uph* mutations (12, 16, 20). Additionally, we identified a set of genes that have not been previously correlated to fosfomycin resistance. Deletion of selected genes in the WT strain led to increased MICs of fosfomycin but not for other classes of antibiotics, indicating that their function is indispensable only for maintaining susceptibility to fosfomycin (Table 2; Table S1). Some mutants, e.g., Δ*purB*, Δ*hflD*, Δ*mutL*, Δ*mutS*, outcompete the WT strain at relatively high drug concentrations (i.e., 32 µg/mL) in competitive and non-competitive assays (Fig. 1 and 2; Fig. S5). These findings highlight the potential adaptive advantage conferred by these mutations under selective pressure, emphasizing the critical role of these genes in shaping bacterial fitness during fosfomycin treatment. Even though the fosfomycin MICs of these mutants are still below the resistance breakpoints set by EUCAST (S ≤ 8 µg/mL) (18) and CLSI (S ≤ 64 µg/mL) (19), mutations in these genes could contribute to clinical resistance phenotypes when combined with other mutations. Thus, we recommend checking the sequence of the genes identified by this study in clinical strains that display resistance in the absence of any known resistance determinants.

A considerable proportion of the mutants selected under high fosfomycin pressure displayed mutations in genes closely associated with central carbon metabolism. These genes encompass all 17 genes within the function categories of phosphotransferase system (PTS) and cAMP regulation, pyruvate metabolism, and the pentose phosphate pathway (Table 1). Rearrangement of metabolic routes and flux is an adaptive strategy of bacteria to evolve tolerance or resistance to antibiotic stress (21). Fosfomycin exerts its antibacterial effect by acting as an analog of phosphoenolpyruvate (PEP), competing with it for binding to MurA (3). PEP is also a vital intermediate in glycolysis (22); thus, it is presumable that interfering with specific metabolic reactions can alter the production and allocation of PEP, thereby modulating the effectiveness of fosfomycin. This is evidenced by previous findings showing that inactivation of central metabolic enzymes reduced fosfomycin susceptibility in *E. coli* (15, 17) and *Stenotrophomonas maltophilia* (23). Additionally, two genes, *plsX* and *fruK*, involved in phospholipid metabolism and fructose metabolism, respectively, also contributed to fosfomycin susceptibility. Even though the function of PlsX in *E. coli* has not been fully characterized yet, it appears to be important for regulating the intracellular concentration of acyl-acyl carrier protein (acyl-ACP), which is the donor of acyl for the G3P acylation step in phospholipid biosynthesis (24). We hypothesize that inactivation of *plsX* leads to acyl-ACP accumulation, which, in turn, accelerates the metabolic flux of G3P acylation, resulting in a decrease in intracellular G3P levels and downregulation of the GlpT transporter. When fructose is utilized as carbon source, FruK specifically catalyzes fructose-1-phosphate to fructose-1,6-biphosphate, which routes to glycolysis as an important intermediate metabolite (25). Reduced fosfomycin susceptibility via *fruK* deletion is possibly attributed to the involvement of fructose PTS system in fosfomycin transport, supported by decreased intracellular concentration of fosfomycin observed in an *E. coli fruK* mutant (26).

Other important biological processes such as nucleotide biosynthesis, protein translation, and assembly of cofactors (e.g., biotin and iron-sulfur cluster) were shown to be essential for maintaining full fosfomycin susceptibility in UPEC (Tables 1 and 2). This corroborates the findings of transcriptomic studies in *Staphylococcus aureus* and *S. maltophilia* showing that the pathways for these processes were downregulated in response to fosfomycin (27, 28). Notably, we observed predominant involvement of the *hflD-purB* operon, in which *hflD* encodes for regulator for lysogenization of bacteriophage λ (29), while *purB* encodes for the key enzyme catalyzing two reactions in *de novo* purine nucleotide biosynthesis (30). Our results suggested that the decreased fosfomycin susceptibility of the Δ*hflD* mutant was due to polar effect on *purB* (Fig. S7; Table S2), while how *purB* contributes to fosfomycin susceptibility requires further investigation. A previous study has shown that deletion of *purB* drastically decreased the level of ATP (31), which is the substrate for cAMP synthesis. It is presumable that inactivation of *purB* reduces cellular cAMP levels, which in turn downregulates the expression of the GlpT and UhpT transporters.

The significant selective advantages and elevated fosfomycin MICs observed in the Δ*mutL* and Δ*mutS* mutants suggest that the DNA mismatch repair system plays a key role in maintaining fosfomycin susceptibility in UPEC (Fig. 1; Table 2). This system repairs the spontaneous mutations that arise during DNA replication (32). Bacteria generate mutations at a higher rate when genes involved in this process (e.g., *mutS* and *mutL*) are inactivated, leading to accumulation of mutations that are advantageous for surviving fosfomycin stress. Intriguingly, deletion of *mutS* or *mutL* in the WT strain resulted in increase of fosfomycin MIC by 16-fold in the presence of G6P, but only 2- to 4-fold increase in the absence of G6P, supporting the notion that G6P may enhance the mutation frequency to fosfomycin resistance (20). This finding apparently contradicts the previous TraDIS study by Turner et al., who stated that knock-out mutations in DNA repair genes are disadvantageous for growth in the presence of fosfomycin (7). However, the output data of the TraDIS analyses provided in the Supplementary Data set of this article show that mutations in DNA repair genes exhibited selective advantages, which is consistent with our findings.

In addition to canonical limitations of TraDIS, such as inability to access essential genes and overlooking essential regions in non-essential genes (33, 34), another major limitation of our study is that our TraDIS experiments were conducted in a single strain. Thus, the findings of our study may not be generalized to different genomic backgrounds and lineages. For example, two previous studies employing an advanced TraDIS approach identified that transposon insertions in the *pstSCAB* genes coding for the phosphate ABC transporter provided selective advantages for growth of *E. coli* K12 BW25113 in the presence of fosfomycin (7, 8), whereas this finding was not confirmed by our data, possibly due to strain-specific factors. We have shown similar differences between laboratory and clinical *E. coli* strains in a previous study on macrolide resistance (35).

In conclusion, this study expands the knowledge of the complex genetic basis of fosfomycin susceptibility in *E. coli*. We identified novel mutations that confer selective advantages to growth and survival of UPEC in the presence of fosfomycin, highlighting the contribution of cellular processes (e.g., central metabolism, nucleotide synthesis, and DNA repair) and metabolic genes (e.g., *purB*) that were not previously associated with fosfomycin resistance.

## MATERIALS AND METHODS

### Strains and reagents

Strains used in this study are listed in Table S3. The clinical UPEC strain, FREC5, was isolated from patients sampled at Copenhagen University Hospital—Copenhagen in 2021. The strain and its derivative mutants were grown at 37°C in Luria-Bertani broth (LB) (Becton Dickinson, Albertslund, Denmark) or on Luria agar (LA) plates. Mueller-Hinton Broth (MHB) (Oxoid, UK) was used for antimicrobial susceptibility testing. Super Optimal broth with Catabolites repression (SOC) medium (Sigma-Aldrich, USA) was used for recovery of competent cells after transformation. Antibiotics including fosfomycin, amoxicillin, trimethoprim, ciprofloxacin gentamicin, azithromycin, kanamycin, chloramphenicol, and tetracycline were purchased from Sigma-Aldrich, USA. EZ-Tn <KAN-2>Tnp Transposome Kit were purchased from LGC Biosearch Technologies. Primers were synthesized in TAG Copenhagen and are listed in Table S4.

### Whole-genome sequencing and analysis

The genome of FREC5 was sequenced by a combination of short- and long-read sequencing. For short-read sequencing, genomic DNA was extracted from overnight culture using the Maxwell RSC Cultured Cells DNA Kit (Promega, Wisconsin, USA), following the manufacturer’s instructions in the Maxwell RSC machine (Promega), and then quantified by Qubit 2.0 Fluorometer (Thermo Fischer Scientific, USA). DNA libraries were constructed by using the Nextera XT library preparation kit (Illumina, USA) following the manufacturer’s protocol, with subsequent sequencing on the MiSeq platform (Illumina, USA). For long-read sequencing, high-molecular-weight DNA extraction was performed using the Monarch HMW DNA Extraction Kit for Tissue (New England Biolabs, Massachusetts, USA) following the manufacturer’s instructions. Nanopore library preparation was performed using the Rapid Barcoding Kit 96, SQK-RBK110-96 (Oxford Nanopore Technologies, Oxford, UK). Sequencing was performed on an Mk1C MinION platform on a Flow Cell R9.4.1 (Oxford Nanopore Technologies).

Raw reads from Illumina sequencing and Nanopore sequencing were assembled using Unicycler v.0.5.0 (36). Plasmids were identified by using PlasmidFinder v.2.1 (https://cge.food.dtu.dk/services/PlasmidFinder/) and were further confirmed by BLASTn analysis against the non-redundant nucleotide NCBI database. Antibiotic resistance genes were identified by using ResFinder v.4.3.3 (http://genepi.food.dtu.dk/resfinder). Chromosomal sequence was annotated by using PROKKA v.1.14.6 (37). All the genes were annotated based on the orthologs from the *E. coli* K-12 substr. MG1655, NCBI RefSeq assembly GCF_000005845.2.

### Construction of TraDIS library

Competent cells were prepared as previously described (38) with slight modifications. In brief, overnight cultures were 1:100 diluted in LB medium. To enhance the electrotransformation efficiency, polymyxin B nonapeptide (Sigma-Aldrich, USA) in a final concentration of 2 µg/mL was additionally supplemented to the LB medium (35). Cells were grown to an OD_600_ of 0.5–0.6, then harvested and washed once with ice-cold water and twice with 10% glycerol, and finally re-suspended in 10% cold glycerol. Then, 1 µL EZ-Tn5 <KAN-2>Tnp Transposome was transformed into an aliquot of competent cells via electroporation. After electroporation, cells were recovered in 1 mL SOC medium for 1.5 h at 37°C and subsequently selected on LA plates supplemented with 50 µg/mL kanamycin. Colonies were collected into MHB medium supplemented with 20% glycerol and then stored at −80°C. To check the saturation of the constructed libraries, genomic DNA were in duplicate extracted from library aliquot containing ~4 × 10^9^ Tn-mutant cells and then processed to TraDIS sequencing and analysis.

### Fosfomycin exposure and TraDIS sequencing

In duplicate, approximately 1 × 10^8^ Tn-mutant cells were inoculated into 10 mL MHB medium supplemented with fosfomycin in concentrations of 0µg/mL, 4 µg/mL, and 32 µg/mL, respectively. Mutants were grown at 37°C for 24 h, and then 1 mL of bacterial culture was used for genomic DNA extraction. Two micrograms of extracted DNA in 100 µL H_2_O was sheared to an average fragment size of 300–400 bp using a sonication device (Bioruptor Pico, Diagenode) with the following profile: 10 cycles of 30 s ON, 90 s OFF at low frequency. The subsequent steps of DNA library preparation was performed following the protocol described in the TraDIS Toolkit method (39), with TraDIS adapter and primers previously designed (39, 40). The resulting DNA was sequenced on a Illumina MiSeq platform using a MiSeq reagent kit V2 (50 cycles) (Illumina, USA), as previously described (39).

### TraDIS data analysis

TraDIS Sequence reads were first processed by using a custom *fq2bam.pl* script to take the transposon tag from each read and add it to the front of the read and then convert the file into SAM format (40). The produced SAM file was subsequently converted into BAM format by using Samtools v.1.19.2 (40). The next steps were performed following the Bio::TraDIS pipeline (https://github.com/sanger-pathogens/Bio-Tradis) (39). In brief, processed reads were mapped to the reference genome of FREC5 to determine read counts and unique transposon insertion sites per gene. Reads in the 10% of the 3′ end of each gene were trimmed out, as many essential genes appear to tolerate insertions toward the end of the coding sequence (39). Statistical analysis was carried out using R v.3.2.3 included in the Bio::Tradis pipeline. Gene essentiality was evaluated using the *tradis_essentiality.R* script, which employs a statistical analysis establishing a bimodal distribution of insertion indexes (IIDs) (number of insertions per gene divided by gene length) for non-essential-genes (gamma) and essential-genes (exponential). Log_2_ likelihood ratios (LLR) were calculated between the fitted distributions, and a gene was classified as essential if showing a LLR < −2, leading to an essentiality cutoff at an IID of 0.0086 in our data. The logFC of read counts and *q*-value for each gene between each two samples were evaluated by using the *tradis_comparisons.R* script. Genes with significantly enriched transposon insertions were selected by cutoff as logFC ≥2 and *q*-value ≤0.05, followed by a manual filter step of checking transposon insertion distribution on Artemis genome browser (https://github.com/sanger-pathogens/Artemis) to exclude those genes that contain high sequencing read counts on a single insertion.

### Construction of gene deletion mutants

Genes of interest were individually deleted in FREC5 by employing the lambda red recombineering as previously described (41), with minor modifications. A Chl^R^ cassette flanked by upstream and downstream homologies to the adjacent chromosomal sequences of the target gene was PCR amplified from pKD3 plasmid and then purified by gel extraction. The temperature-sensitive lambda red recombinase plasmid pKD46-Gm (42) was transformed into FREC5 via electroporation. Strains carrying the pKD46-Gm plasmid were incubated in LB medium supplemented with 20 µg/mL gentamicin and shaken at 28°C overnight and then 1:100 diluted into fresh LB medium supplemented with 20 µg/mL gentamicin and 2 µg/mL polymyxin B nonapeptide. L-arabinose (Sigma-Aldrich, USA) at a final concentration of 80 mM was added to the subculture when the OD_600_ reached 0.4. After 1.5 h of induction, the cells were made electrocompetent and then introduced the Chl^R^ cassette by electroporation. Recombined mutants were selected on LA plates containing 20 µg/mL chloramphenicol at 37°C and then verified by colony PCR using primers listed in Table S4.

### Gene complementation

A low-copy-number plasmid, pACYC184, was used for gene complementation to mutants of interest. Briefly, the backbone of pACYC184 and the coding sequence of the target gene were PCR amplified using primers listed in Table S4. After DNA purification, the plasmid backbone and gene insert were assembled by using the NEBuilder HiFi DNA Assembly Master Mix (New England Biolabs) according to the manufacturer’s protocol. The resulted complementary plasmid was transformed into the mutant and transformants were selected on LA plates containing 10 µg/mL tetracycline and verified by colony PCR. The original pACYC184 plasmid was transformed to the WT strain and the mutants as controls.

### Antimicrobial susceptibility testing

MIC of fosfomycin was tested by using the agar dilution method on MHA plate supplemented with 25 µg/mL of G6P (Sigma-Aldrich) following the CLSI protocol (19). Consistent MIC values from three biological replicates are reported. *E. coli* ATCC 25922 was used as control strain.

### Growth curve assay

In triplicates, overnight MHB culture of a single strain, or aliquots of transposon mutant library, were diluted to ~2 × 10^6^ cells in 200 µL fresh MHB medium supplemented with or without fosfomycin in wells of a 96-well microtiter plate. The prepared plate was covered with lid and placed in a microplate reader (BioTek). Absorbance at 600 nm (OD_600_) in each well was measured every 15 min in a 24 h duration by setting a kinetic measurement with continuous orbital shaking at 37°C.

### Competitive fitness assays

In triplicates, overnight MHB cultures of the FREC5 WT strain and mutants were diluted to 0.5 McFarland standards which was comparable to a cell density of ~1.5 × 10^8^ colony-forming unit (CFU) per ml. For each mutant, 50 µL of the dilution was mixed with 50 µL of the WT dilution. The 100 µL of mixture was then added into 9.9 mL of MHB medium supplemented with or without fosfomycin at a final concentration of 32 µg/mL. After 6, 12, and 24 h of incubation at 37°C, the co-cultures of WT strain and mutant were serially diluted and then spotted onto LA plates and LA plates supplementing 10 µg/mL of chloramphenicol (LAC) separately. After another 24 h of incubation at 37°C, colonies on the LA plates were counted for the CFUs of the entire bacterial population containing both the WT strain and the mutant, while colonies on the LAC plates for solely the mutant.

## Data Availability

Whole genome sequence of UPEC strains and TraDIS sequencing reads have been submitted to the NCBI Sequence Read Archive (SRA) under BioProject accession no. PRJNA1162300.

## SUPPLEMENTAL MATERIAL

Supplemental figures and tables aac.01417-24-s0001.docx Figures S1 to S7; Tables S1 to S4.

Supplemental data set aac.01417-24-s0002.xlsx Genes exhibiting increase in mutant frequencies under different experimental conditions.

ASM does not own the copyrights to Supplemental Material that may be linked to, or accessed through, an article. The authors have granted ASM a non-exclusive, world-wide license to publish the Supplemental Material files. Please contact the corresponding author directly for reuse.

## References

[1] Gupta K, Hooton TM, Naber KG, Wullt B, Colgan R, Miller LG, Moran GJ, Nicolle LE, Raz R, Schaeffer AJ, Soper DE, Infectious Diseases Society of America, European Society for Microbiology and Infectious Diseases. 2011. International clinical practice guidelines for the treatment of acute uncomplicated cystitis and pyelonephritis in women: a 2010 update by the Infectious Diseases Society of America and the European Society for Microbiology and Infectious Diseases. Clin Infect Dis 52:e103–20. doi:10.1093/cid/ciq257 21292654

[2] Ejrnæs K. 2011. Bacterial characteristics of importance for recurrent urinary tract infections caused by Escherichia coli. Dan Med Bull 58:B4187. 21466767

[3] Silver LL. 2017. Fosfomycin: mechanism and resistance. Cold Spring Harb Perspect Med 7:a025262. doi:10.1101/cshperspect.a025262 28062557 PMC5287057

[4] Castañeda-García A, Blázquez J, Rodríguez-Rojas A. 2013. Molecular mechanisms and clinical impact of acquired and intrinsic fosfomycin resistance. Antibiotics (Basel) 2:217–236. doi:10.3390/antibiotics2020217 27029300 PMC4790336

[5] Takahata S, Ida T, Hiraishi T, Sakakibara S, Maebashi K, Terada S, Muratani T, Matsumoto T, Nakahama C, Tomono K. 2010. Molecular mechanisms of fosfomycin resistance in clinical isolates of Escherichia coli. Int J Antimicrob Agents 35:333–337. doi:10.1016/j.ijantimicag.2009.11.011 20071153

[6] Zurfluh K, Treier A, Schmitt K, Stephan R. 2020. Mobile fosfomycin resistance genes in Enterobacteriaceae-An increasing threat. Microbiologyopen 9:e1135. doi:10.1002/mbo3.1135 33128341 PMC7755807

[7] Turner AK, Yasir M, Bastkowski S, Telatin A, Page AJ, Charles IG, Webber MA. 2020. A genome-wide analysis of Escherichia coli responses to fosfomycin using TraDIS-Xpress reveals novel roles for phosphonate degradation and phosphate transport systems. J Antimicrob Chemother 75:3144–3151. doi:10.1093/jac/dkaa296 32756955 PMC7566553

[8] Coward C, Dharmalingham G, Abdulle O, Avis T, Beisken S, Breidenstein E, Carli N, Figueiredo L, Jones D, Khan N, Malara S, Martins J, Nagalingam N, Turner K, Wain J, Williams D, Powell D, Mason C. 2020. High-density transposon libraries utilising outward-oriented promoters identify mechanisms of action and resistance to antimicrobials. FEMS Microbiol Lett 367:fnaa185. doi:10.1093/femsle/fnaa185 33186989 PMC7735965

[9] Cattoir V, Pourbaix A, Magnan M, Chau F, de Lastours V, Felden B, Fantin B, Guérin F. 2020. Novel chromosomal mutations responsible for fosfomycin resistance in Escherichia coli. Front Microbiol 11:575031. doi:10.3389/fmicb.2020.575031 33193186 PMC7607045

[10] Wachino J, Yamane K, Suzuki S, Kimura K, Arakawa Y. 2010. Prevalence of fosfomycin resistance among CTX-M-producing Escherichia coli clinical isolates in Japan and identification of novel plasmid-mediated fosfomycin-modifying enzymes. Antimicrob Agents Chemother 54:3061–3064. doi:10.1128/AAC.01834-09 20404116 PMC2897269

[11] Ríos E, Del Carmen López Diaz M, Culebras E, Rodríguez-Avial I, Rodríguez-Avial C. 2022. Resistance to fosfomycin is increasing and is significantly associated with extended-spectrum β-lactamase-production in urinary isolates of Escherichia coli. Med Microbiol Immunol 211:269–272. doi:10.1007/s00430-022-00749-2 36056943 PMC9618510

[12] Pourbaix A, Guérin F, Lastours V de, Chau F, Auzou M, Boulley E, Cattoir V, Fantin B. 2017. Biological cost of fosfomycin resistance in Escherichia coli in a murine model of urinary tract infection. Int J Med Microbiol 307:452–459. doi:10.1016/j.ijmm.2017.09.019 28986014

[13] Oteo J, Orden B, Bautista V, Cuevas O, Arroyo M, Martínez-Ruiz R, Pérez-Vázquez M, Alcaraz M, García-Cobos S, Campos J. 2009. CTX-M-15-producing urinary Escherichia coli O25b-ST131-phylogroup B2 has acquired resistance to fosfomycin. J Antimicrob Chemother 64:712–717. doi:10.1093/jac/dkp288 19671590

[14] Lucas AE, Ito R, Mustapha MM, McElheny CL, Mettus RT, Bowler SL, Kantz SF, Pacey MP, Pasculle AW, Cooper VS, Doi Y. 2018. Frequency and mechanisms of spontaneous fosfomycin nonsusceptibility observed upon disk diffusion testing of Escherichia coli. J Clin Microbiol 56:e01368-17. doi:10.1128/JCM.01368-17 29093108 PMC5744208

[15] Bermudez TA, Brannon JR, Dudipala N, Reasoner S, Morales G, Wiebe M, Cecala M, DaCosta M, Beebout C, Amir O, Hadjifrangiskou M. 2024. Raising the alarm: fosfomycin resistance associated with non-susceptible inner colonies imparts no fitness cost to the primary bacterial uropathogen. Antimicrob Agents Chemother 68:e0080323. doi:10.1128/aac.00803-23 38078906 PMC10777853

[16] Marchese A, Gualco L, Debbia EA, Schito GC, Schito AM. 2003. In vitro activity of fosfomycin against gram-negative urinary pathogens and the biological cost of fosfomycin resistance. Int J Antimicrob Agents 22 Suppl 2:53–59. doi:10.1016/s0924-8579(03)00230-9 14527772

[17] Hirakawa H, Takita A, Sato Y, Hiramoto S, Hashimoto Y, Ohshima N, Minamishima YA, Murakami M, Tomita H. 2023. Inactivation of ackA and pta genes reduces GlpT expression and susceptibility to fosfomycin in Escherichia coli. Microbiol Spectr 11:e0506922. 37199605 10.1128/spectrum.05069-22 PMC10269713

[18] EUCAST. 2024. Breakpoint tables for interpretation of MICs and zone diameters, version 14.0, 2024. The European Committee on Antimicrobial Susceptibility Testing.

[19] CLSI. 2024. Performance standards for antimicrobial susceptibility testing, 34th ed. In CLSI supplement M100. Clinical and Laboratory Standards Institute, Wayne, PA.

[20] Nilsson AI, Berg OG, Aspevall O, Kahlmeter G, Andersson DI. 2003. Biological costs and mechanisms of fosfomycin resistance in Escherichia coli. Antimicrob Agents Chemother 47:2850–2858. doi:10.1128/AAC.47.9.2850-2858.2003 12936984 PMC182645

[21] Zampieri M, Enke T, Chubukov V, Ricci V, Piddock L, Sauer U. 2017. Metabolic constraints on the evolution of antibiotic resistance. Mol Syst Biol 13:917. doi:10.15252/msb.20167028 28265005 PMC5371735

[22] Meza E, Becker J, Bolivar F, Gosset G, Wittmann C. 2012. Consequences of phosphoenolpyruvate:sugar phosphotranferase system and pyruvate kinase isozymes inactivation in central carbon metabolism flux distribution in Escherichia coli. Microb Cell Fact 11:127. doi:10.1186/1475-2859-11-127 22973998 PMC3521201

[23] Gil-Gil T, Corona F, Martínez JL, Bernardini A. 2020. The inactivation of enzymes belonging to the central carbon metabolism is a novel mechanism of developing antibiotic resistance. mSystems 5:e00282-20. doi:10.1128/mSystems.00282-20 32487742 PMC8534728

[24] Yoshimura M, Oshima T, Ogasawara N. 2007. Involvement of the YneS/YgiH and PlsX proteins in phospholipid biosynthesis in both Bacillus subtilis and Escherichia coli. BMC Microbiol 7:69. doi:10.1186/1471-2180-7-69 17645809 PMC1950310

[25] Kornberg HL. 2001. Routes for fructose utilization by Escherichia coli. J Mol Microbiol Biotechnol 3:355–359. 11361065

[26] Gil-Gil T, Martínez JL. 2024. Role of the phosphotransferase system in the transport of fosfomycin in Escherichia coli. Int J Antimicrob Agents 63:107027. doi:10.1016/j.ijantimicag.2023.107027 37926273

[27] Petek M, Baebler S, Kuzman D, Rotter A, Podlesek Z, Gruden K, Ravnikar M, Urleb U. 2010. Revealing fosfomycin primary effect on Staphylococcus aureus transcriptome: modulation of cell envelope biosynthesis and phosphoenolpyruvate induced starvation. BMC Microbiol 10:159. doi:10.1186/1471-2180-10-159 20515462 PMC2887449

[28] Gil-Gil T, Ochoa-Sánchez LE, Martínez JL. 2021. The antibiotic fosfomycin mimics the effects of the intermediate metabolites phosphoenolpyruvate and glyceraldehyde-3-phosphate on the stenotrophomonas maltophilia transcriptome. Int J Mol Sci 23:159. doi:10.3390/ijms23010159 35008587 PMC8745565

[29] Kihara A, Akiyama Y, Ito K. 2001. Revisiting the lysogenization control of bacteriophage lambda. Identification and characterization of a new host component, HflD. J Biol Chem 276:13695–13700. doi:10.1074/jbc.M011699200 11278968

[30] Zhang Y, Morar M, Ealick SE. 2008. Structural biology of the purine biosynthetic pathway. Cell Mol Life Sci 65:3699–3724. doi:10.1007/s00018-008-8295-8 18712276 PMC2596281

[31] Sun Y, Fukamachi T, Saito H, Kobayashi H. 2011. ATP requirement for acidic resistance in Escherichia coli. J Bacteriol 193:3072–3077. doi:10.1128/JB.00091-11 21478347 PMC3133219

[32] Putnam CD. 2016. Evolution of the methyl directed mismatch repair system in Escherichia coli. DNA Repair (Amst) 38:32–41. doi:10.1016/j.dnarep.2015.11.016 26698649 PMC4740232

[33] Ma Y, Pirolo M, Jana B, Mebus VH, Guardabassi L. 2024. The intrinsic macrolide resistome of Escherichia coli. Antimicrob Agents Chemother. doi:10.1128/aac.00452-24:e0045224 PMC11304742 38940570

[34] Cain AK, Barquist L, Goodman AL, Paulsen IT, Parkhill J, van Opijnen T. 2020. A decade of advances in transposon-insertion sequencing. Nat Rev Genet 21:526–540. doi:10.1038/s41576-020-0244-x 32533119 PMC7291929

[35] Qin J, Hong Y, Pullela K, Morona R, Henderson IR, Totsika M. 2022. A method for increasing electroporation competence of Gram-negative clinical isolates by polymyxin B nonapeptide. Sci Rep 12:11629. doi:10.1038/s41598-022-15997-8 35804085 PMC9270391

[36] Wick RR, Judd LM, Gorrie CL, Holt KE. 2017. Unicycler: resolving bacterial genome assemblies from short and long sequencing reads. PLoS Comput Biol 13:e1005595. doi:10.1371/journal.pcbi.1005595 28594827 PMC5481147

[37] Seemann T. 2014. Prokka: rapid prokaryotic genome annotation. Bioinformatics 30:2068–2069. doi:10.1093/bioinformatics/btu153 24642063

[38] Green MR, Sambrook J. 2020. Transformation of Escherichia coli by electroporation. Cold Spring Harb Protoc 2020:101220. doi:10.1101/pdb.prot101220 32482901

[39] Barquist L, Mayho M, Cummins C, Cain AK, Boinett CJ, Page AJ, Langridge GC, Quail MA, Keane JA, Parkhill J. 2016. The TraDIS toolkit: sequencing and analysis for dense transposon mutant libraries. Bioinformatics 32:1109–1111. doi:10.1093/bioinformatics/btw022 26794317 PMC4896371

[40] García V, Grønnemose RB, Torres-Puig S, Kudirkiene E, Piantelli M, Ahmed S, Andersen TE, Møller-Jensen J, Olsen JE, Herrero-Fresno A. 2021. Genome-wide analysis of fitness-factors in uropathogenic Escherichia coli during growth in laboratory media and during urinary tract infections. Microb Genom 7. doi:10.1099/mgen.0.000719 PMC8767336 34928200

[41] Datsenko KA, Wanner BL. 2000. One-step inactivation of chromosomal genes in Escherichia coli K-12 using PCR products. Proc Natl Acad Sci U S A 97:6640–6645. doi:10.1073/pnas.120163297 10829079 PMC18686

[42] Doublet B, Douard G, Targant H, Meunier D, Madec JY, Cloeckaert A. 2008. Antibiotic marker modifications of lambda Red and FLP helper plasmids, pKD46 and pCP20, for inactivation of chromosomal genes using PCR products in multidrug-resistant strains. J Microbiol Methods 75:359–361. doi:10.1016/j.mimet.2008.06.010 18619499

