# Supplementary material for: Chromosomal genes modulating fosfomycin susceptibility in uropathogenic Escherichia coli: a genome-wide analysis
Source: Antimicrob Agents Chemother. 2025 Feb 25;69(4):e01417-24. doi: 10.1128/aac.01417-24 (PMC11963563; doi:10.1128/aac.01417-24)
Supplement: Supplemental figures and tables — Figures S1 to S7; Tables S1 to S4. [file aac.01417-24-s0001.docx]

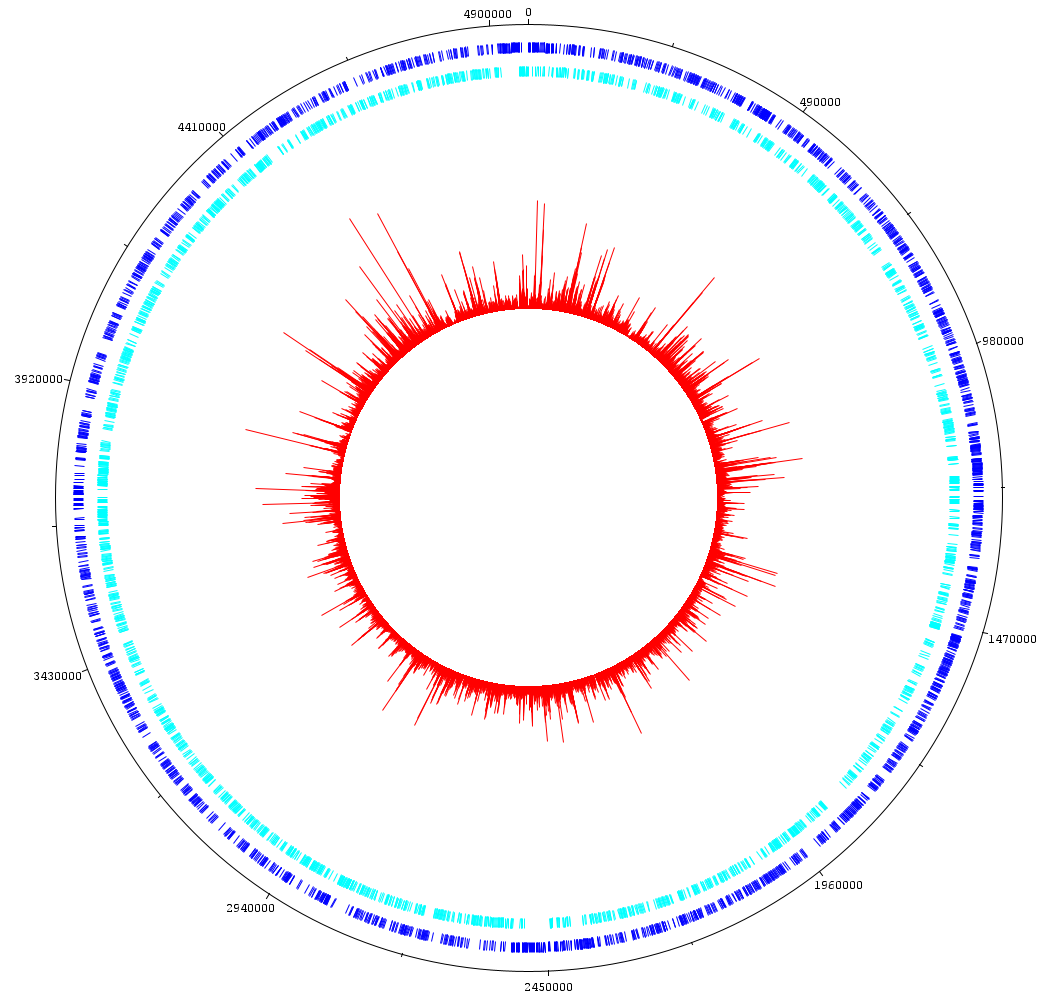


**FIGURE S1**. Genome-wide transposon insertion sites mapped to the genome of uropathogenic *Escherichia coli* FREC5. The black outermost track represents the *E. coli* genome, with base pairs marked from the annotation origin. The next two inner tracks in blue and cyan correspond to the sense and antisense coding DNA sequences (CDS), respectively. The innermost circle displays red lines, indicating the location and frequency of transposon insertions mapped onto the genome. The figure was generated using DNAPlotter (Artemis).

**
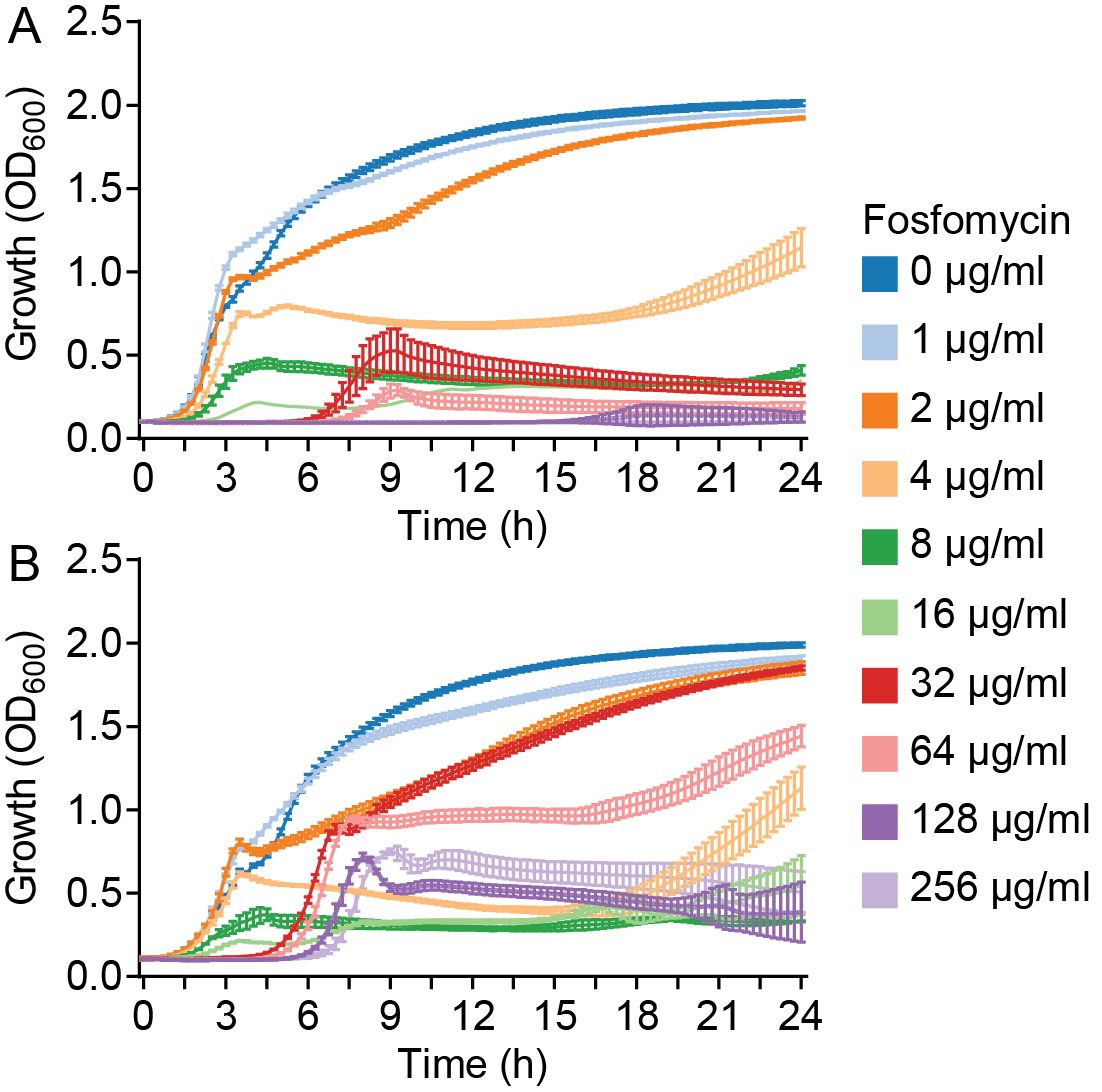
**

**FIGURE S2** Growth kinetics of the wild-type strain (A) and transposon mutant library (B) of FREC5. Bacteria were grown in MHB media supplemented with fosfomycin at concentrations ranging from 0 to 256 µg/ml. Growth (OD_600_) was measured at every 15 minutes in 24 h. Three biological replicates were included for each sample and data represents the mean ± standard deviation of the triplicates.


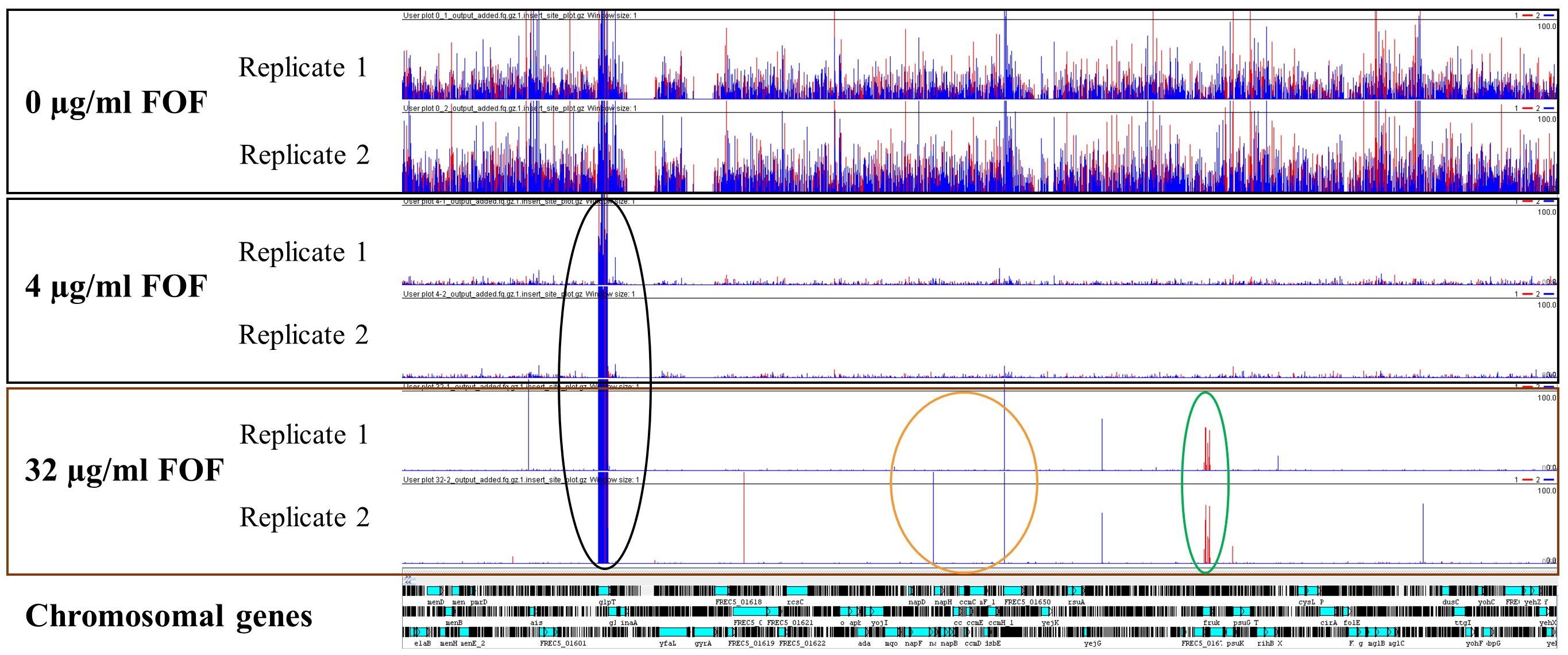


**FIGURE S3**. Transposon insertion map of FREC5 genome segment. Insertion site reads were plotted on the chromosomal sequence and inspected using the Artemis genome browser. Lines in red or blue color indicate the transposon insertion in two orientations. Different patterns of insertion enrichment at certain genes following fosfomycin (FOF) exposure are marked by ovals, including enrichment at both 4 µg/ml and 32 µg/ml (black), enrichment only at 32 µg/ml (green), and enrichment at a single insertion site (orange).

**
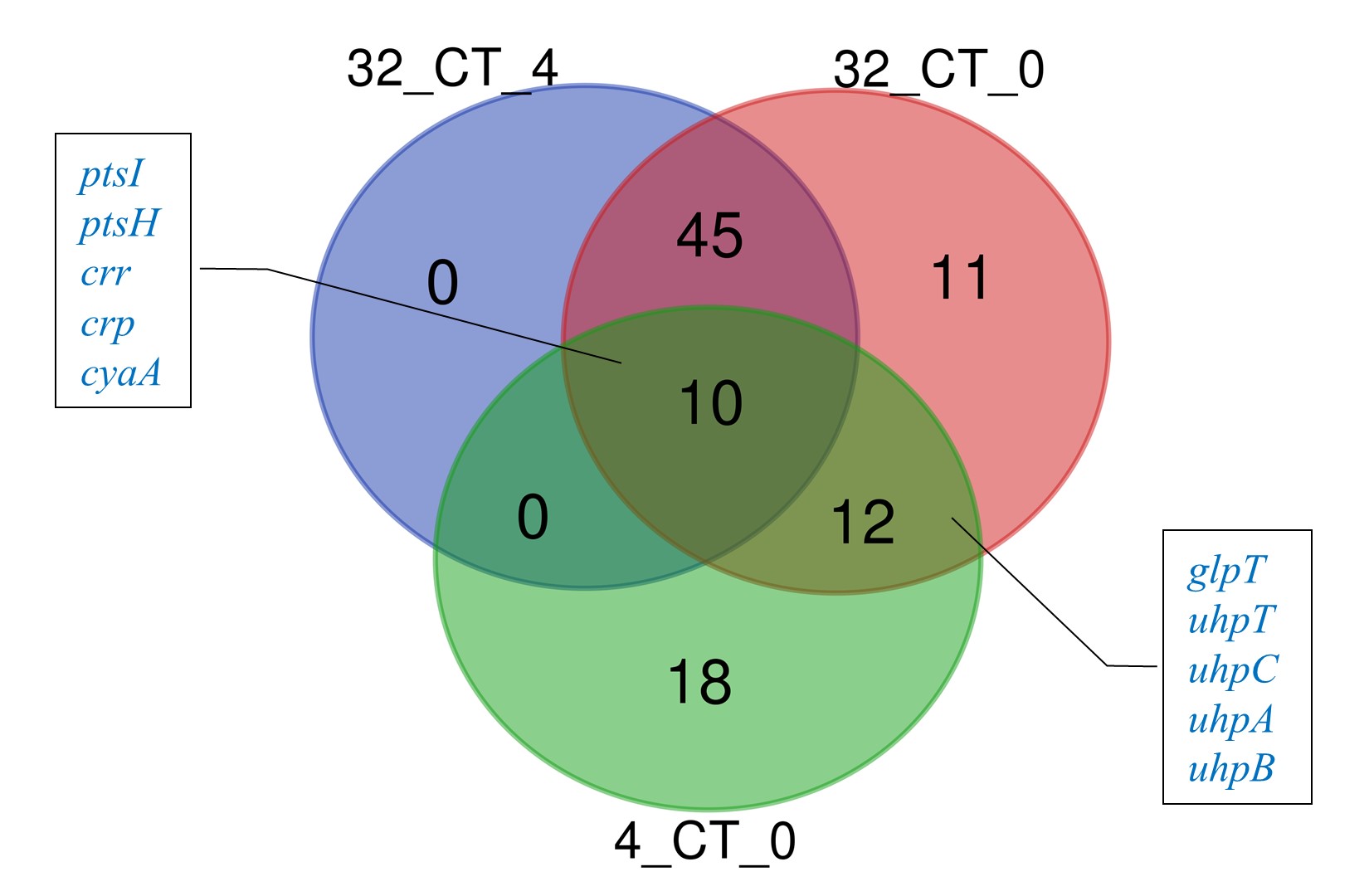
**

**FIGURE S4** Venn-diagram of genes with transposon insertion frequencies significantly enriched at 32 µg/ml compared to 4 µg/ml (32_CT_4), 32 µg/ml compared to 0 µg/ml (32_CT_0), and 4 µg/ml compared to 0 µg/ml (4_CT_0). Genes related to known fosfomycin resistance mechanisms are shown in callouts.

**
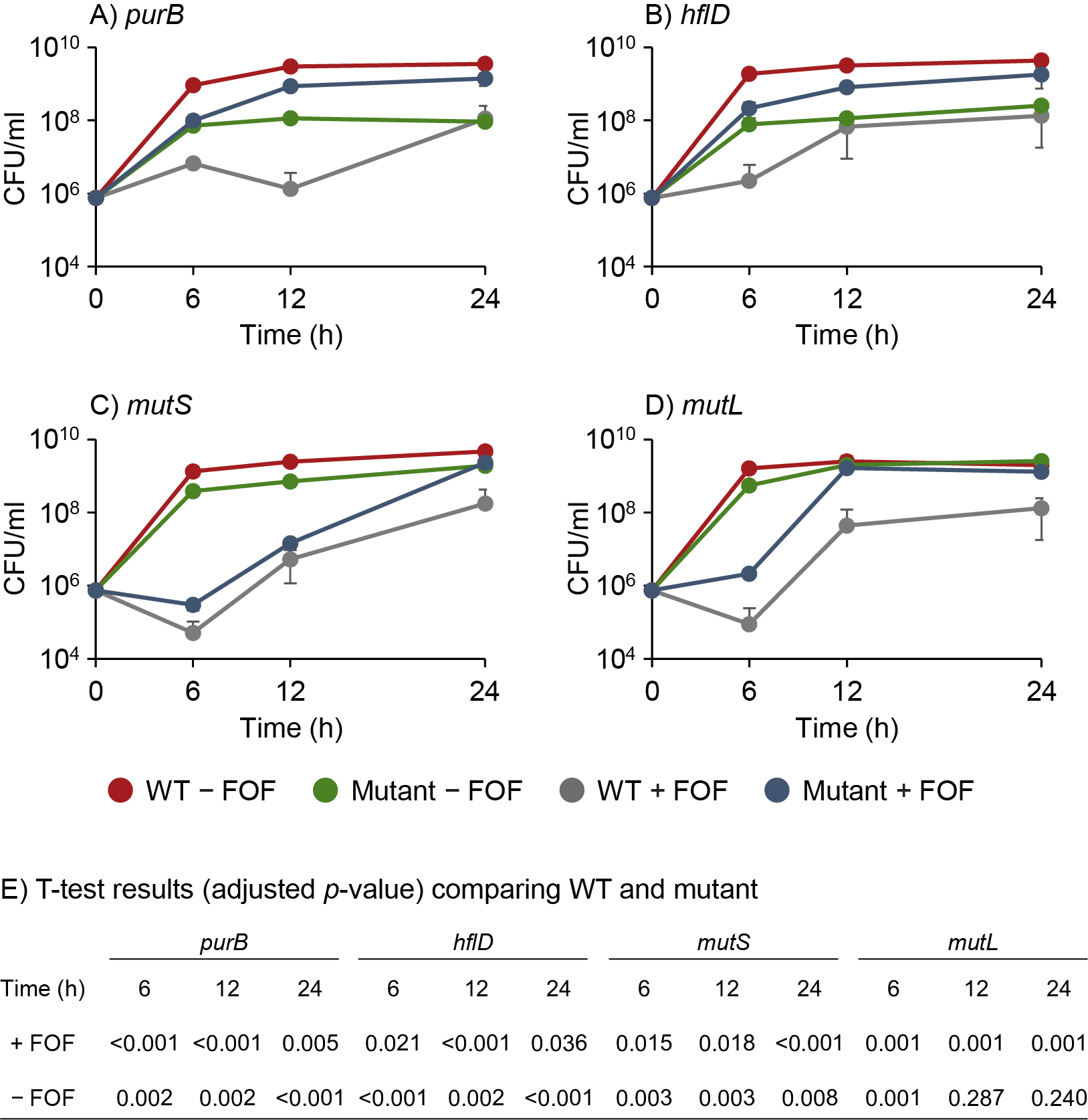
**

**FIGURE S5** Fitness competition between gene-deletion mutants and the wild-type strain (WT) of FREC5 in the absence and presence of 32 µg/ml of fosfomycin (FOF). WT was co-cultured with Δ*purB* (A), Δ*hflD* (B), Δ*mutS* (C) or Δ*mutL* (D) for 6, 12 and 24 h, followed by colony-forming unit (CFU) counting at each time point. Three biological replicates were included for each sample and data represents the mean ± standard deviation of the triplicates. (E) FDR-adjusted *p*-values from t-test comparing WT and mutants in the absence and presence of 32 µg/ml of FOF across time points.

**
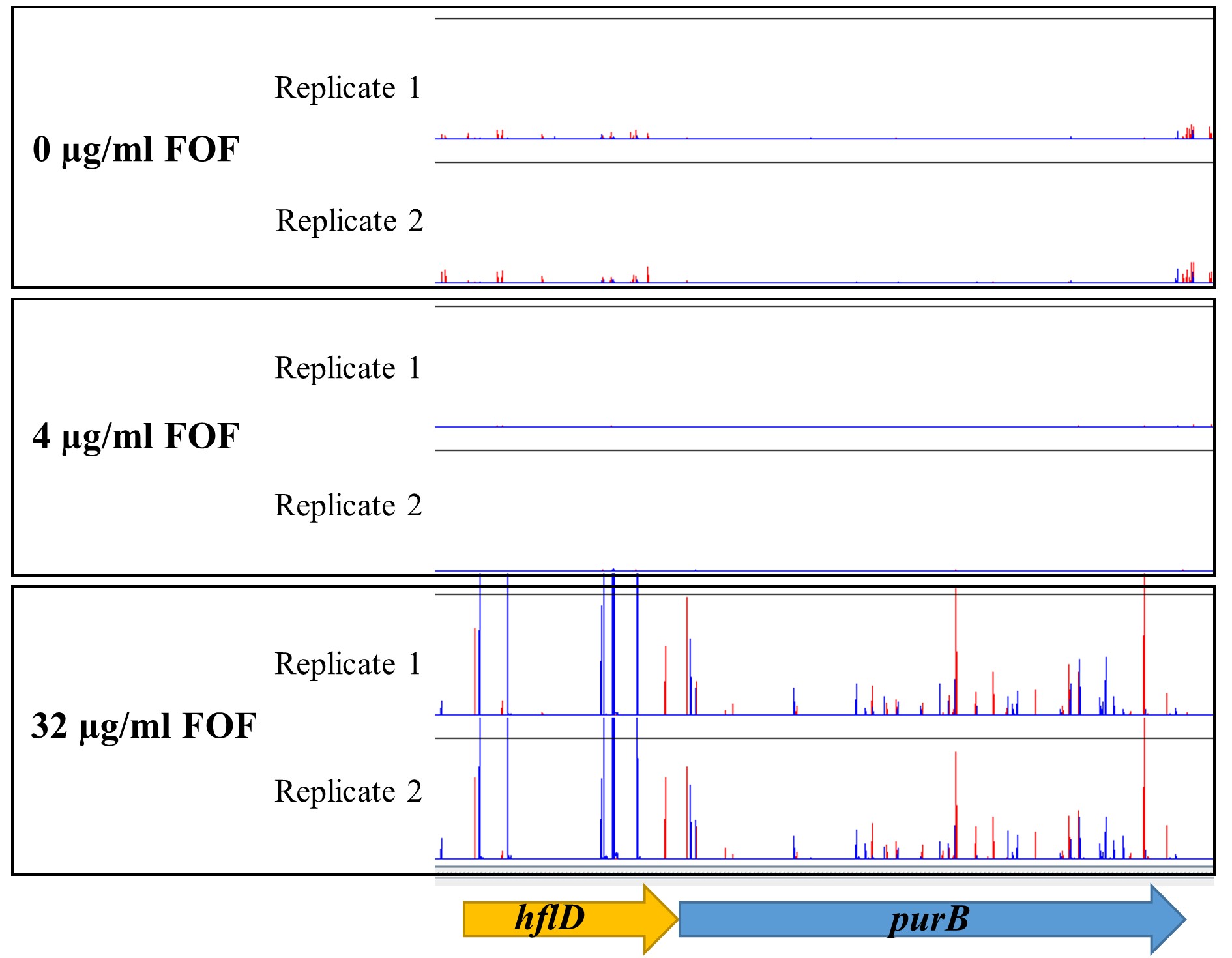
**

**FIGURE S6** Transposon insertion map of the *hflD*-*purB* operon following fosfomycin (FOF) exposure at 0, 4, or 32 µg/ml. Insertion site reads were plotted on the gene sequences and inspected using the Artemis genome browser.

**
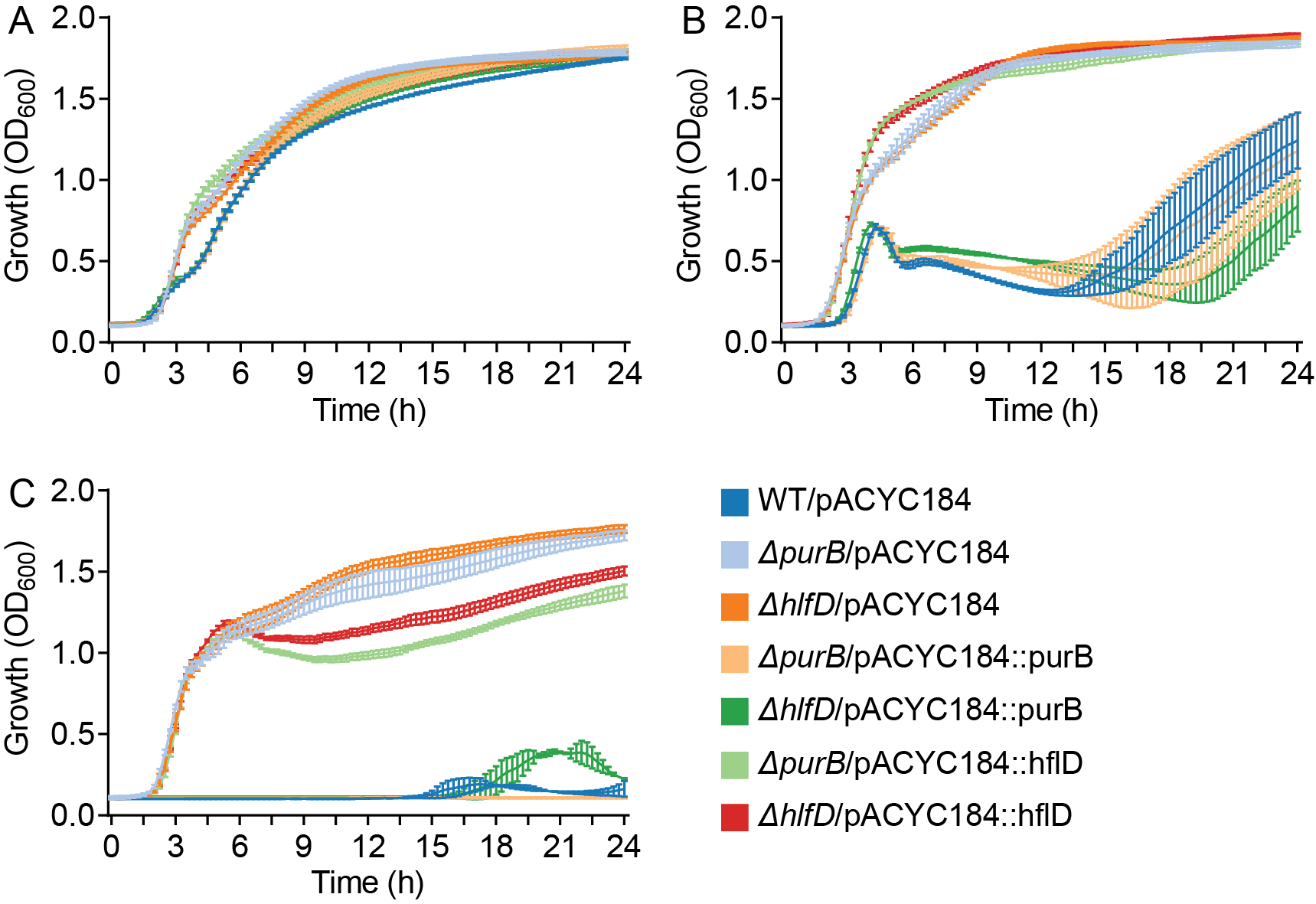
FIGURE S7** Growth curves of FREC5 wild-type (WT) strain, mutant and complementation strains of *purB* and *hflD* in MHB media supplemented with 0 µg/ml (A), 4 µg/ml (B) or 32 µg/ml (C) of fosfomycin. Growth (OD_600_) was measured at every 15 minutes in 24 h. Three biological replicates were included for each sample and data represents the mean ± standard deviation of the triplicates.

**TABLE S1** MICs (µg/ml) of amoxicillin (AMX), trimethoprim (TMP), ciprofloxacin (CIP), gentamycin (GEN) and azithromycin (AZI) in the wild-type and gene-deletion mutants of FREC5

| **Genotype** | **AMX** | **TMP** | **CIP** | **GEN** | **AZI** |
| --- | --- | --- | --- | --- | --- |
| WT | 2 | 0.5 | 0.016 | 2 | 8 |
| Δ*mutL* | 2 | 0.5 | 0.03 | 2 | 8 |
| Δ*mutS* | 2 | 0.5 | 0.03 | 4 | 16 |
| Δ*hflD* | 2 | 0.125 | 0.03 | 0.5 | 4 |
| Δ*purB* | 1 | 0.125 | 0.016 | 0.5 | 4 |
| Δ*plsX* | 2 | 1 | 0.016 | 2 | 4 |
| Δ*pdhR* | 2 | 0.25 | 0.016 | 1 | 8 |
| Δ*aceF* | 2 | 1 | 0.03 | 1 | 4 |
| Δ*rng* | 1 | 0.5 | 0.016 | 1 | 4 |
| Δ*rpe* | 2 | 0.25 | 0.016 | 1 | 4 |
| Δ*tktA* | 2 | 0.25 | 0.016 | 1 | 4 |
| Δ*yceD* | 1 | 0.5 | 0.03 | 1 | 4 |
| Δ*bioC* | 1 | 0.25 | 0.016 | 0.25 | 4 |
| Δ*bioD* | 1 | 0.25 | 0.016 | 0.25 | 4 |
| Δ*hscB* | 2 | 0.5 | 0.016 | 2 | 4 |
| Δ*fur* | 1 | 0.5 | 0.03 | 1 | 2 |
| Δ*fruK* | 1 | 0.25 | 0.016 | 1 | 4 |

**TABLE S2** Fosfomycin MICs (µg/ml) in the wild-type (WT), mutant and complementation strains of *purB* and *hflD* in FREC5 tested with or without supplement of 25 mg/l glucose-6-phosphate (G6P)

| **Genotype** | **MIC with G6P** | **MIC without G6P** |
| --- | --- | --- |
| WT/pACYC184 | 0.5 | 64 |
| Δ*purB/*pACYC184 | 2 | 512 |
| Δ*hflD/*pACYC184 | 2 | 512 |
| Δ*purB/*pACYC184::*purB* | 0.5 | 64 |
| Δ*hflD/*pACYC184::*purB* | 0.5 | 128 |
| Δ*purB/*pACYC184::*hflD* | 2 | 256 |
| Δ*hflD/*pACYC184::*hflD* | 2 | 256 |

**TABLE S3** List of strains used in the study.

| **Strains** | **Description** | **Reference** |
| --- | --- | --- |
| FREC5 | A clinical uropathogenic *E. coli* strain; ESBL-negative; ST7093 | This study |
| ATCC25922 | *E. coli* reference strain for antimicrobial susceptibility testing | CLSI^*^ |
| FREC5 Δ*mutL* | *mutL* deletion mutant of FREC5; CHL^R^ | This study |
| FREC5 Δ*mutS* | *mutS* deletion mutant of FREC5; CHL^R^ | This study |
| FREC5 Δ*hflD* | *hflD* deletion mutant of FREC5; CHL^R^ | This study |
| FREC5 Δ*purB* | *purB* deletion mutant of FREC5; CHL^R^ | This study |
| FREC5 Δ*plsX* | *plsX* deletion mutant of FREC5; CHL^R^ | This study |
| FREC5 Δ*pdhR* | *pdhR* deletion mutant of FREC5; CHL^R^ | This study |
| FREC5 Δ*aceF* | *aceF* deletion mutant of FREC5; CHL^R^ | This study |
| FREC5 Δ*rng* | *rng* deletion mutant of FREC5; CHL^R^ | This study |
| FREC5 Δ*rpe* | *rpe* deletion mutant of FREC5; CHL^R^ | This study |
| FREC5 Δ*tktA* | *tktA* deletion mutant of FREC5; CHL^R^ | This study |
| FREC5 Δ*yceD* | *yceD* deletion mutant of FREC5; CHL^R^ | This study |
| FREC5 Δ*bioC* | *bioC* deletion mutant of FREC5; CHL^R^ | This study |
| FREC5 Δ*bioD* | *bioD* deletion mutant of FREC5; CHL^R^ | This study |
| FREC5 Δ*hscB* | *hscB* deletion mutant of FREC5; CHL^R^ | This study |
| FREC5 Δ*fur* | *fur* deletion mutant of FREC5; CHL^R^ | This study |
| FREC5 Δ*fruK* | *fruK* deletion mutant of FREC5; CHL^R^ | This study |
| FREC5/pACYC184 | FREC5 transformed with pACYC184 plasmid; CHL^R^; TET^R^ | This study |
| Δ*purB/*pACYC184 | FREC5 Δ*purB* transformed with pACYC184 plasmid; CHL^R^; TET^R^ | This study |
| Δ*hflD/*pACYC184 | FREC5 Δ*hflD* transformed with pACYC184 plasmid; CHL^R^; TET^R^ | This study |
| Δ*purB/*pACYC184::*purB* | FREC5 Δ*purB* transformed with pACYC184 plasmid carrying *purB*; CHL^R^; TET^R^ | This study |
| Δ*hflD/*pACYC184::*purB* | FREC5 Δ*hflD* transformed with pACYC184 plasmid carrying *purB*; CHL^R^; TET^R^ | This study |
| Δ*purB/*pACYC184::*hflD* | FREC5 Δ*purB* transformed with pACYC184 plasmid carrying *hflD*; CHL^R^; TET^R^ | This study |
| Δ*hflD/*pACYC184::*hflD* | FREC5 Δ*hflD* transformed with pACYC184 plasmid carrying *hflD*; CHL^R^; TET^R^ | This study |

^*^CLSI, Clinical and Laboratory Standards Institute; Performance standards for antimicrobial susceptibility testing, 34th ed. CLSI supplement M100; Wayne, PA.

**TABLE S4** List of primers used in the study

| **Name** | **Description** | **Sequence** **(5'-3')** |
| --- | --- | --- |
| purB_KO_F | Forward primer for *purB* deletion | CCACTCAGGCAAAACAAATTCTTGCTCATTTAACCCCGGAGTTGTGATCTGTGTAGGCTGGAGCTGCTTC |
| purB_KO_R | Reverse primer for *purB* deletion | AATTTTAATAAACAGGCCGGACAGCATCGCCATCCGGCACTGATACGAGTATGGGAATTAGCCATGGTCC |
| hflD_KO_F | Forward primer for *hflD* deletion | CCGCTGCCGGTCTGATTATTATCTTTACTTAAAACAAGGAAGCAGTGAACGTGTAGGCTGGAGCTGCTTC |
| hflD_KO_R | Reverse primer for *hflD* deletion | TAGCGTCCATCGACAGGGGAAACGGCGGTCAGTGAGGATAATTCCATAGAATGGGAATTAGCCATGGTCC |
| yceD_KO_F | Forward primer for *yceD* deletion | CTGCCTTTTTCTTTGACTCTATGACGTTACAAAGTTAATATGCGCGCCCTGTGTAGGCTGGAGCTGCTTC |
| yceD_KO_R | Reverse primer for *yceD* deletion | GACCTTACTCCTCAATTACGGTTTATCCCCGATCCAACGGGGAGCACCAAATGGGAATTAGCCATGGTCC |
| plsX_KO_F | Forward primer for *plsX* deletion | AACCGGGCGGCGACGATACCTTGACACGTCTAACCCTGGCGTTAGATGTCGTGTAGGCTGGAGCTGCTTC |
| plsX_KO_R | Reverse primer for *plsX* deletion | TCGGTTATATACCGTCACTTGCAAACTGCGAGTTCGCTGGCAGCGTCCTGATGGGAATTAGCCATGGTCC |
| rpe_KO_F | Forward primer for *rpe* deletion | AAACCAGGAGTCGTTTCACCCGCGAAAAAATAATTCTCAAGGAGAAGCGGGTGTAGGCTGGAGCTGCTTC |
| rpe_KO_R | Reverse primer for *rpe* deletion | GATCAAAAGCGACGCCGCGAATATCTTCAAACTTATTCATGACTTACCTTATGGGAATTAGCCATGGTCC |
| tktA_KO_F | Forward primer for *tktA* deletion | GTCGTCAAGTCGTTAAGGGCGTGCCCTTCATCATCCGATCTGGAGTCAAAGTGTAGGCTGGAGCTGCTTC |
| tktA_KO_R | Reverse primer for *tktA* deletion | CAAGGTAATAAAAAAGGTCGCCGAAGCGACCTTTTTACCCGAAATGCTAAATGGGAATTAGCCATGGTCC |
| pdhR_KO_F | Forward primer for *pdhR* deletion | GAAATTGGTAAGACCAATTGACTTCGGCAAGTGGCTTAAGACAGGAACTCGTGTAGGCTGGAGCTGCTTC |
| pdhR_KO_R | Reverse primer for *pdhR* deletion | TATGCGCTTGATTTACAACATCTTCTGGATAATTTTTACCAGAAAAATCAATGGGAATTAGCCATGGTCC |
| aceF_KO_F | Forward primer for *aceF* deletion | ATCGATGCAGATAAAGTTAACCCGCGTCTGGCGTAAGAGGTAAAAGAATAGTGTAGGCTGGAGCTGCTTC |
| aceF_KO_R | Reverse primer for *aceF* deletion | CATTCATGAGATTACCAGAAAAAAGCCGGCCGTTGGGCCGGCTCTTTTACATGGGAATTAGCCATGGTCC |
| rng_KO_F | Forward primer for rng deletion | TTAACGCACTGCGTGAGAAAAGGGATAAACATGACGGCTGAATTGTTAGTGTGTAGGCTGGAGCTGCTTC |
| rng_KO_R | Reverse primer for *rng* deletion | CTAAAAATGTGACTCAAAAACCCTTTGCCGGATGGCGGCCCAGCATCTGTATGGGAATTAGCCATGGTCC |
| fruK_KO_F | Forward primer for *fruK* deletion | AATCGGCGACGCTATCGCTGCTGGTCTTGGGGAGGGCGCATAATGAGCAGGTGTAGGCTGGAGCTGCTTC |
| fruK_KO_R | Reverse primer for *fruK* deletion | CGAGATTAGCGTCAATAATCAGCAGCGTTTTCATTATGCCTCTCCTGCTGATGGGAATTAGCCATGGTCC |
| mutS_KO_F | Forward primer for *mutS* deletion | AATTTCGACGCCCATACGCCCATGATGCAGCAGTATCTCAAGCTGAAAGCGTGTAGGCTGGAGCTGCTTC |
| mutS_KO_R | Reverse primer for *mutS* deletion | GTCAGTTGTCGTTAATATTCCCGATAGCAAAAGACTATCGGGAATTGTTAATGGGAATTAGCCATGGTCC |
| bioC_KO_F | Forward primer for *bioC* deletion | ATCGACCGTCTGCTGGAGGTGCTGCATGGTAACGGTTAATAAACAAGCCAGTGTAGGCTGGAGCTGCTTC |
| bioC_KO_R | Reverse primer for *bioC* deletion | TCGGTATCCGTTCCGGTGACAAAATAACGTTTACTCACGAGCAATCACTCATGGGAATTAGCCATGGTCC |
| bioD_KO_F | Forward primer for *bioD* deletion | CGTATCATCTTTTTTTGGGAGTGATTGCTCGTGAGTAAACGTTATTTTGTGTGTAGGCTGGAGCTGCTTC |
| bioD_KO_R | Reverse primer for *bioD* deletion | AGTCAATCGAAGACGCGATCTCGCTCGCAATTTAACCAAATACAGGATAGATGGGAATTAGCCATGGTCC |
| hscB_KO_F | Forward primer for *hscB* deletion | GTTTTACCTGATTCGCCGTTATCGCGGCGGATCGCAGCCCTGAGAATGTTGTGTAGGCTGGAGCTGCTTC |
| hscB_KO_R | Reverse primer for *hscB* deletion | TCAAACCAGGTTCACTAATTTGTAATAAGGCCATGTTTAGCTTCCAGAAAATGGGAATTAGCCATGGTCC |
| fur_KO_F | Forward primer for *fur* deletion | TGTCACTTCTTCTAATGAAGTGAACCGCTTAGTAACAGGACAGATTCCGCGTGTAGGCTGGAGCTGCTTC |
| fur_KO_R | Reverse primer for *fur* deletion | TTGCATAAAAAAGCCAACCCGCAGGTTGGCTTTTTCTCATTCAGGCTGGCATGGGAATTAGCCATGGTCC |
| mutL_KO_F | Forward primer for *mutL* deletion | CCAGTACGGTGACGACGCCAGATCGCACGCTGCCAAACTAAGGACGATTGGTGTAGGCTGGAGCTGCTTC |
| mutL_KO_R | Reverse primer for *mutL* deletion | ACAGACCACCCGGCGGCGTTTTCACAAGTTGCGGACATAACCGTTCCACGATGGGAATTAGCCATGGTCC |
| hflD-purB_veri_F | Forward primer for Δ*hflD* and Δ*purB* verification | CGGTGGCGGTATTATTGAGC |
| hflD-purB_veri_R | Reverse primer for Δ*hflD* and Δ*purB* verification | GTCGTGGTTAGTGGTACGCT |
| yceD-plsX_veri_F | Forward primer for Δ*yceD* and Δ*plsX* verification | CCCGCAATGTGTGCGAATTA |
| yceD-plsX_veri_R | Reverse primer for Δ*yceD* and Δ*plsX* verification | CTGACTGCGCAGGAATAATC |
| rpe_veri_F | Forward primer for Δ*rpe* verification | CGCAGTATAAGCAGCAACGG |
| rpe_veri_R | Reverse primer for Δ*rpe* verification | GGCAACTCCAGTGCATACAG |
| tktA_veri_F | Forward primer for Δ*tktA* verification | GGCTTGCGGTAAATTGTTGG |
| tktA_veri_R | Reverse primer for Δ*tktA* verification | CGCTTTATCTATTGCTTGTCCG |
| pdhR_veri_F | Forward primer for Δ*pdhR* verification | GTTGTTAAAATGTGCACAGTTTC |
| pdhR_veri_R | Reverse primer for Δ*pdhR* verification | GAGTTTTCTGGAACCTGTCC |
| aceF_veri_F | Forward primer for Δ*aceF* verification | AACGTGGCGAAATCGATAAG |
| aceF_veri_R | Reverse primer for Δ*aceF* verification | GGTCGTTCTATCCGTCGGTA |
| rng_veri_F | Forward primer for Δ*rng* verification | GATAAATGGCAGCTATCACG |
| rng_veri_R | Reverse primer for Δ*rng* verification | CAGTAAAATCCCCGGCAATC |
| fruK_veri_F | Forward primer for Δ*fruK* verification | CGTTAAGAAAGGTCATCGCC |
| fruK_veri_R | Reverse primer for Δ*fruK* verification | TCTGCGTCGTTCGGATTGTC |
| mutS_veri_F | Forward primer for Δ*mutS* verification | CCATCACACCCCATTTAATATC |
| mutS_veri_R | Reverse primer for Δ*mutS* verification | GATAGCGTGTAGATGGCATG |
| bioCD_veri_F | Forward primer for Δ*bioC* and Δ*bioD* verification | CAACCGTACCCGCTGGTACT |
| bioCD_veri_R | Reverse primer for Δ*bioC* and Δ*bioD* verification | CAATTCCCACATTCGCCGCA |
| hscB_veri_F | Forward primer for Δ*hscB* verification | GTTGCGGCGAAAGCTTCCAC |
| hscB_veri_R | Reverse primer for Δ*hscB* verification | GTTGTGCCCAGGTCAATACC |
| fur_veri_F | Forward primer for Δ*fur* verification | CATTTAGGCGTGGCAATTCT |
| fur_veri_R | Reverse primer for Δ*fur* verification | GGTGAAAGCCGATTGTGATA |
| mutL_veri_F | Forward primer for Δ*mutL* verification | CCAACAACAGCTGGCAGAAG |
| mutL_veri_R | Reverse primer for Δ*mutL* verification | GCAGGCTCGCCTTACTGATA |
| pACYC184_F | Forward primer for amplification of pACYC184 backbone | TTTTTTTAAGGCAGTTATTGGTGCC |
| pACYC184_R | Reverse primer for amplification of pACYC184 backbone | TTTAGCTTCCTTAGCTCCTGAAAAT |
| pACYC184_veri_F | Forward primer for pACYC184 verification | CACTTCCCTGTTAAGTATCT |
| purB_C_F | Forward primer for amplification of *purB* complementation | CAGGAGCTAAGGAAGCTAAAATGGAATTATCCTCACTGAC |
| purB_C_R | Reverse primer for amplification of *purB* complementation | CAATAACTGCCTTAAAAAAATTATTTCAGCTCATCAACCA |
| purB_C_veri_R | Reverse primer for verification of *purB* complementation | GTCGTGGTTAGTGGTACGCT |
| hflD_C_F | Forward primer for amplification of *hflD* complementation | CAGGAGCTAAGGAAGCTAAAGTGGCAAAGAATTACTATGA |
| hflD_C_R | Reverse primer for amplification of *hflD* complementation | CAATAACTGCCTTAAAAAAATCACAACTCCGGGGTTAAAT |
| hflD_C_veri_R | Reverse primer for verification of *hflD* complementation | CAAGCACCATCAAGCTGAGT |
